# Campbell systematic reviews takes next step to meeting FAIR principles

**DOI:** 10.1002/cl2.1032

**Published:** 2019-07-05

**Authors:** Vivian A. Welch

**Affiliations:** ^1^ Campbell Collaboration

With this first double issue of Campbell Systematic Reviews launching our publishing partnership with John Wiley & Sons, the Campbell Collaboration moves one step closer to adhering to the FAIR principles (Findable, Accessible, Interoperable, Reusable) of research (https://www.force11.org/group/fairgroup/fairprinciples). The FAIR principles are central to ensuring reproducibility, transparency, and accountability in science.

Since its beginning in 2001, the Campbell Collaboration has held the principle of accessibility at its heart through open‐access publication of both the plan for the review (the protocol) and the full review. We publish peer‐reviewed, transparent rigorous systematic reviews in the social sciences as well as methodological guidance. With Wiley, we will continue to publish open access, using a CC‐BY 4.0 licences where authors retain the copyright to their own material. We will also continue to welcome copublication with specialty journals which reach the specialty practitioners and policy‐makers such as police departments, teachers, and schoolboards. One example is our long‐standing relationship with the Research in Social Work Practice journal.

Findability and discoverability was a key deciding factor in our motivation to partner with Wiley in January 2019. In our first year, Wiley will ensure indexing in relevant social science databases as well as supporting our communication of review findings within its global social science networks, particularly in Asia. So far, we have documented over 20 policy influence stories of how Campbell systematic reviews have been used in policy documentation and legislation (https://campbellcollaboration.org/blog/must‐try‐harder‐policy‐influence‐from‐campbell‐reviews.html). Altmetrics enabled through the Wiley platform will allow us to identify additional press and media stories as well as citations in policy documents. Wiley will also ensure a permanent archive of all content.

Interoperability refers to the ability for data to be used in different ways, for example, by making meta‐data such as included references and tables machine readable. The entire Campbell Systematic Reviews collection will be published on the Wiley platform as full‐text .html, which will allow the content of each article to be more easily searched, and will also facilitate searching of meta‐data. In 2017, Campbell decided to transition to using Revman as its authoring tool, generously provided by the Cochrane Collaboration, a long‐time and valued partner. Revman provides a consistent structure for systematic reviews and has an efficient way to ensure version control for multiple author systematic reviews.

Reusability is linked to interoperability since the data needs to be interoperable to be reusable. However data also needs to be shared for reusability. Repositories for systematic review data already exist such as the SR data repository (Brown University) and will soon be available within the R metafor package. These repositories enable sharing of not only outcome data but also coding of study characteristics such as methods, population and risks of bias. In the coming years, we encourage Campbell authors to make their data available on platforms such as these, ideally where the data can be cited.

Priorities for the next year include:

1 Expanding article types to better address questions from our communities of practitioners and policy‐makers, including qualitative evidence synthesis, evidence and gap maps, reviews of reviews, methods research studies and guidance. We are also developing editor tools and training to build capacity in these new article types.
2 Advancing relevant methods standards. For example, we are forming a working group to consider what types of nonrandomized studies, how to synthesize with randomized trials and how to appraise risk of bias. We also expect our qualitative evidence synthesis working group to finalize draft guidance this fall.
3 Improving efficiency, transparency, and accountability of systematic reviews. This will involve trialing automation in relevant steps of the systematic review process, with human verification. We also continue to aim to prevent wasteful overlap or duplication of prior systematic reviews, while at the same time recognizing that replication of systematic reviews may be valuable for policy and practice, for example, to confirm findings, test assumptions or understand reasons for controversy. We are collaborating on policy guidance on when to replicate and when not to replicate systematic reviews with an international working group which will be available in early 2020. We are part of a common movement for greater research transparency, and plan to make more of the (meta)data from our reviews available.

I am confident that our move to publish with Wiley and our focus on supporting the publication of high quality, leading‐edge systematic reviews that are relevant for policy and practice will help improve our impact on social policies and the lives of people affected by these policies.

Join us in this exciting period of innovation! I would love to hear your ideas to make Campbell systematic reviews faster, more robust and better fit for purpose. Let's produce better evidence for a better world together.

John Wiley & Sons Limited is a private limited company registered in England with registered number 641132. Registered office address: The Atrium, Southern Gate, Chichester, West Sussex, United Kingdom. PO19 8SQ.

